# Betulinic Acid–Doxorubicin-Drug Combination Induced Apoptotic Death via ROS Stimulation in a Relapsed AML MOLM-13 Cell Model

**DOI:** 10.3390/antiox10091456

**Published:** 2021-09-14

**Authors:** Milan Vu, Nick Kassouf, Sandra Appiah

**Affiliations:** Department of Natural Sciences, Faculty of Science and Technology, Middlesex University, London NW4 4BT, UK; M.vu@mdx.ac.uk (M.V.); N.Kassouf@mdx.ac.uk (N.K.)

**Keywords:** acute myeloid leukaemia, doxorubicin, betulinic acid, apoptosis, drug combination, B-cell lymphoma 2 family of proteins, reactive oxygen species

## Abstract

In this study, cell death regulation and induction in AML cell line from a relapsed MLL-rearranged cell model (MOLM-13) was investigated with doxorubin (Dox) and betulinic acid (BetA), singly and in combination. CyQUANT Direct^®^ and Annexin V/propidium iodide double staining were used to measure the cytotoxic and cell death induction effects of the compounds, respectively. Reactive oxygen species (ROS) generation was measured using 2′,7′-dichlorofluorescin diacetate staining. Expressions of proteins and genes were examined by Western blot and reverse transcription polymerase chain reaction analysis, respectively. BetA (20 μM) and Dox (1 μM) indicated a synergistic growth inhibitory effect on MOLM-13 cells. The combined drug caused more cells to reside in irreversible late apoptotic stage compared to the single treatments (*p* < 0.05). Elevation in ROS may be the synergistic mechanism involved in MOLM-13 cell death since ROS can directly disrupt mitochondrial activity. In contrast, in leukaemic U-937 cells, the combination treatments attenuated Dox-induced cell death. Dox and the drug combination selectively reduced (*p* < 0.05) a recently reported anti-apoptotic Bcl-2 protein isoform p15-20-Bcl-2 in MOLM-13 by our group, without affecting the usually reported p26-Bcl-2-α. Further studies using known inhibitors of apoptosis are required to confirm the potential of Dox-BetA combination to modulate these pathways.

## 1. Introduction

Betulinic acid (3β,hydroxyl-lup-20(29)-en-28-oic acid, BetA), pentacyclic triterpenoid, has been reported to be effective against relapsed acute myeloid leukaemia (AML) cells when tested alongside standard AML chemotherapeutics such as doxorubicin (Dox) and cytarabine (tested at clinically relevant concentrations) [1]. Furthermore, it has been reported that BetA exerts some selectivity towards cancer cells whilst being less toxic towards non-cancerous cells. This effect has been described in both blood cells and cells of epithelial origin, such as human primary melanocytic cells [2], normal colon cells [3], peripheral blood mononuclear cells [4], and normal peripheral blood lymphoblasts [5]. Therefore, the compound could be a good candidate for drug combination studies with standard chemotherapy treatments as it has been suggested to enhance drug-induced cell death or sensitize the cancer cells to a drug [6]. Most studies have reported that BetA-induced cell death in cancer cells is through apoptotic death, specifically by affecting the intrinsic (mitochondrial) pathway [1,7,8,9]. However, due to inconsistent reports between different cell types on the role of Bcl-2 family regulation by BetA [10,11,12,13], further studies are warranted to examine BetA’s mechanism of multiple drug interaction and synergism.

Apoptosis is a programmed cell death pathway that is usually the primary target of most chemotherapy drugs [14]. For instance, Dox, an established AML drug [15], works as a topoisomerase II inhibitor [16,17] and has been reported to induce apoptotic cell death in various cancers [18,19,20,21,22]. This cell death signalling is regulated via extrinsic or intrinsic apoptotic pathways, which leads to proteolytic events that activate enzyme caspases to execute cell death [23,24]. Apoptosis through the extrinsic pathway is triggered by death receptors present on the cell surface while the intrinsic pathway is activated by mitochondrial perturbation [25,26]. Mitochondrial membrane permeability is regulated by reactive oxygen species (ROS), as well as by B-cell lymphoma 2 (Bcl-2) family of proteins, which are either pro-apoptotic (Bax, Bak, Bad) or anti-apoptotic (Bcl-2, Bcl-XL, Mcl-1). These proteins contain BH3 domain that can form a binding groove, which allows them to interact and facilitate or antagonise each function [27]. Some cancers, including AML cells abnormally regulate the Bcl-2 family of proteins [28,29,30,31], which could contribute to their refractory nature in relapsed incidents.

Some cancer cells can develop chemoresistance by stimulating autophagy [32]. This cellular process is tightly linked to apoptosis through Beclin 1:Bcl-2 complex regulation, where Bcl-2 can suppress the autophagic function of Beclin 1 [33]. This is because Beclin 1 also contains a BH3 domain and is essential in initiating the phagophore formation of the autophagosome [33]. Autophagy is a dynamic cell process that initiates cell survival/protection, as well as promoting cell death [25,32], thus modulating this pathway could be useful in fighting cancerous cells [33,34]. Cellular stress triggers this signalling pathway, which leads to formation of isolation membranes and vesicles that recycle aged cellular content [26,34,35].

We have recently published findings of a possible Bcl-2 variant, p15-20-Bcl-2 in AML MOLM-13 cells, which was selectively targeted by Dox [22]. This currently reported study aimed to determine if combining BetA and Dox could enhance the cytotoxicity of Dox to MOLM-13 cells.

## 2. Materials and Methods

### 2.1. Cell Culture

An authenticated leukaemic MOLM-13 cell line (immortalized AML cell line derived from a relapsed patient with MLL-rearrangement) was purchased from the European Collection of Cell Cultures, Public Health England and maintained in Roswell Park Memorial Institute (RPMI) 1640 (Sigma-Aldrich; Merck KGaA, UK) medium. The AML U-937 (ATCC CRL-1593.2) cell lines were purchased from American Type Culture Collection. U-937 cells were grown and maintained in Iscove’s Modified Dulbecco’s medium (Sigma-Aldrich; Merck KGaA, UK). This study compares two AML cell lines of relapsed (MOLM-13) and non-relapsed (U-937) origin with different mutations.

Mycoplasma-free cells (tested by polymerase chain reaction) were used throughout the experiments. The culture mediums were further supplemented with 1% L-glutamine, 1% penicillin-streptomycin antibiotic and 10% foetal bovine serum (Sigma-Aldrich; Merck KGaA, UK) and the cells were grown in a humidified incubator at 37 °C in 5% CO_2_ environment. The medium was replenished every 48–72 h through cell pelleting by centrifugation. Experiments were done on cells with a viability of at least 97% in logarithmic growth, which was tested by Trypan blue (HyClone^®^, South Logan, UT, USA) dye assay to determine cell density. Cells were manually counted at 1:1 ratio with the dye using a haemocytometer under light microscopy.

### 2.2. Drugs and Treatment Concentrations

BetA 20 μM was selected as a suitable concentration for combination studies since BetA 10 μg/mL (22 μM) has been reported to be non-toxic in human cells in in vitro studies [1,36]. This concentration is relative to 100 mg/kg administration and found to be non-toxic in in vivo studies [37]. The Dox concentrations chosen were within 0.1–1 μM, a range that is equivalent to clinical concentrations [38]. Dox 0.5 μM and 1 μM were selected concentrations for combination with BetA 20 μM. 

To determine the non-cytotoxic concentration range of DMSO suitable for solubilising the test drugs, the effect of DMSO (0.05% to 1%) on various cell lines was investigated in preliminary experiments (data not shown). Cell viability was assessed by CyQUANT Direct^®^. Up to 0.07%, DMSO was found to be non-toxic; 0.05% DMSO was selected as the final concentration for the dissolution of test compounds, maintaining the same level of DMSO concentration in all the treatments, including those for the negative and positive controls. 

Stock solutions (20× the final conc.) of betulinic acid (BetA; Sigma-Aldrich; Merck KGaA, Gillingham, UK) and doxorubicin (Dox; Sigma-Aldrich; Merck KGaA, UK) were prepared in 1% DMSO (in medium or PBS) and the cell treatments were conducted in 1:20 dilution, with a final concentration of 0.05% DMSO in all treatments. The final concentrations of the drugs in cell treatments were as follows: BetA (20 μM), Dox (0.5 μM, 1 μM) or a combination of both BetA and Dox was added. 

The effect of the treatments on the cells was investigated by using two different approaches, by cell viability with CyQUANT Direct^®^ and by differentiation of the population of live and dead cells through Annexin V and PI staining. The cell health in CyQUANT Direct^®^ assessment was only by the level of nucleic acid of viable cells in cell treatments relative to the control. Cell death population by Annexin V/PI was displayed in the ratio of live cells and different cell death (early apoptosis, late apoptosis, and necrotic cells).

### 2.3. Determination of Cell Viability and Cytotoxicity Using CyQUANT Direct^®^ Assay

MOLM-13 and U-937 cells were seeded at 5 × 10^5^ cells/mL in 96-well plates, treated (with single or combined treatments of BetA and Dox; Section 2.2), and incubated for 24 h. Cells treated with the DMSO vehicle (final conc. 0.05%) were used as negative control. After the treatment period, CyQUANT Direct^®^ (Invitrogen, Thermo Fisher Scientific; Loughborough, UK) dye was overlaid in each well (in a 96-well plate) and incubated for a further 45 min (in a humidified incubator at 37 °C) to determine the viability of the cells. CyQUANT^®^ detection dye was prepared by mixing nucleic acid stain (0.4%), background suppressor I (2%), and culture media (97.6%). CyQUANT dye stains nucleic acids (RNA and DNA) in dividing cells and is used as an indicator of cell number since DNA/RNA content is highly and tightly regulated in live cells [39,40]. To compensate for drug (particularly for Dox) Dox auto-fluorescence, individual blank controls (drug + diluent) were used for each sample to subtract background fluorescence. The fluorescence of the samples was measured using a microplate reader FLUOstar Omega (BMG Labtech) at 1000 gain at 485 nm excitation and 520 nm emission wavelength.

### 2.4. Cell Death Population Assays Using 488 Annexin V and PI: Flow Cytometry

MOLM-13 and U-937 cells (1 × 10^6^ cells/mL) were treated with BetA, Dox, or BetA combined with Dox (see Section 2.2) for 24 and 48 h in 5% CO_2_ and at 37 °C. Cells treated with 0.05% DMSO vehicle were used as positive and negative controls, respectively. As previously described [22], flow cytometry analysis was used to differentiate viable and dead (early/late apoptosis and necrosis) cells using Alexa Fluor^®^ 488 Annexin V (Thermo Fisher Scientific) method. The cell events of live and dead populations were characterised by quadrant separation and presented as mean percentage ratio of three biological replicates. The difference in the cell population (live and dead) was compared between the control and treatments.

### 2.5. Reactive Oxygen Species (ROS) Formation

DCFDA cellular ROS detection assay (Abcam, Cambridge, UK) was used to measure ROS activity in cells using the fluorogenic dye 2′,7′-dichlorofluorescin diacetate (DCFDA). The oxidation product, 2′,7′–dichlorofluorescein is highly fluorescent and was measured to estimate ROS levels in the cells. Harvested MOLM-13 cell lines were first washed in cold PBS followed by 1× buffer wash. The cells were then stained by suspending them in DCFDA solution (20 µM) at a concentration of 1 × 10^6^ cells/mL and incubated at 37 °C for 30 min in the dark. After incubation, the cells were washed in 1× buffer and re-suspended at 1 × 106 cells/mL in 1× supplement buffer (1× buffer containing 10% FBS) and seeded at 95 µL/well in a 96-well microplate. Cells were then treated by BetA, Dox, or BetA combined with Dox (see Section 2.2) and incubated at 37 °C in 5% CO_2_. Tert-butyl hydrogen peroxide (TBHP) (final conc. 50 µM) and Dox (final conc. 5 μM) were used as the positive control drugs, and the vehicle (DMSO final conc. 0.05%) as the negative control. In addition, drug-treated but unstained cells and untreated cells were also used as controls. The fluorescence of 2′,7′–dichlorofluorescein was measured at 30 min intervals up to 3.5 h using a FLUOstar Omega microplate reader (BMG Labtech) at 1000 gain, 485 nm excitation and 520 nm emission wavelengths.

### 2.6. Investigation of Proteins Involved in Cell Death: Western Blot Analysis

#### 2.6.1. Cell Treatment

The cells were treated with BetA, Dox, DMSO (final conc. 0.05%; vehicle control) and two combinations of BetA (final conc. 20 μM) with Dox (final conc. 0.5 or 1 μM). The treatments were incubated for 48 h at 37 °C in humidified atmosphere of 5% CO_2_. Whole cell lysis, protein SDS-PAGE separation, membrane transfer, immunoblotting and visualisation were performed following methods that were previously described in detail [22].

#### 2.6.2. Antibodies

The primary antibodies used were as follows: anti-Bcl-2 (1:1000 in 5% milk; Abcam cat. No. ab32124), anti-Bax (1:1000 in 5% milk; Abcam cat. No. ab32503), and anti-Beclin 1 (1:2000 in 5% BSA; Abcam cat. No. ab207612). All were probed with a secondary antibody Goat Anti-Rabbit IgG (H + L) horseradish peroxidase (1:3000 in 5% BSA or 5% milk; BIO-RAD cat. No. 170-6515). Primary antibody for β-actin (1:5000 in 5% BSA; Abcam cat. No. ab8226) was used as a housekeeping protein control, which was probed by another secondary antibody, Goat Anti-Mouse IgG (H + L) horseradish peroxidase-labelled secondary antibody (1:3000 in 5% BSA or 5% milk; BIO-RAD cat. No. 170-6516).

### 2.7. Investigating Gene Regulation via RT-PCR

#### 2.7.1. Cell Treatment and RNA Isolation

MOLM-13 and U-937 cells at 1 × 10^6^ cells/mL density were treated with BetA, Dox, or combination treatment of BetA (final conc. 20 µM, 25 µL) with Dox (final conc. 1 µM, 25 µL) for 48 h, with DMSO (final con. 0.05%) as a vehicle control. Prior to RNA isolation, the cells were pelleted via centrifugation, snap frozen in liquid nitrogen and stored at −80 °C. The RNA samples were obtained using a silica membrane binding method utilised within the Isolate II RNA mini assay (Bioline, London, UK). The RNA concentration and purity were checked using a Nanodrop (NanoDrop 2000/2000c, Thermo Scientific, Loughborough, UK).

#### 2.7.2. Reverse Transcription–Polymerase Chain Reaction (RT-PCR)

A standard amount of RNA at 200 ng, for all samples, was converted to cDNA by reverse transcription and amplified by PCR utilising a one-step RT-PCR method; the MyTaqTM One-Step RT-PCR Kit (Bioline, UK) in a thermal cycler (Techne^®^ Prime, Bibby Scientific, Stone, UK). Isolated RNA was prepared in a final volume of 25 µL in a mixture containing 12.5 µL 2× MyTaq One-Step Mix, 0.25 µL reverse transcriptase, 0.5 µL RiboSafe RNase Inhibitor (10 U/µM), and 1 µL of respective forward and reverse primers. Specific volume of Diethyl Pyrocarbonate (DEPC) H_2_O was used to dilute and standardise the RNA template to 200 ng. Samples with the same mixture but without the RNA template was used as non-template control. The RT-PCR conditions on the thermocycler were as follows: reverse-transcription (1 cycle, 45 °C, 20 min), polymerase activation (1 cycle, 95 °C, 1 min), denaturation (30 cycles, 95 °C, 10 s), annealing (30 cycles, 60 °C, 10 s), and extension (30 cycles, 72 °C, 30 s). Specific primers for pro-apoptotic BAK and BAX, anti-apoptotic BCL-2 and BCL-XL, autophagy marker ATG5 and BECLIN 1 (Table 1; all from Invitrogen, Thermo Fisher Scientific) and for the housekeeping gene GAPDH (Invitrogen, Thermo Fisher Scientific) (Fwd: ACCACAGTCCATGCCATCAC; Rev: TCCACCACCCTGTTGCTGTA) were used for gene targeting. An intercalating DNA binding stain, SafeView (5 μL/100 mL agarose), was added to the 1.5% agarose gel. PCR products were loaded into the gel and the electrophoresis was carried out at a constant 90 V for 30 min. The gel image was visualised under UV illumination using an UVP GelDoc-It^®^ imaging system 2UV transilluminator. Densitometry was carried out using ImageJ software (Madison, WI, USA).

### 2.8. Data and Statistical Analysis

The data was analysed using Minitab 18^®^ software (State College, PA, USA) for statistical comparison. The data was expressed as a mean percentage ± SE relative to the vehicle control from a sample size of at least three replicates. The data was analysed using (independent) two sample *t*-test (comparison of two means) or one-way ANOVA (comparison of multiple means) after performing Anderson–Darling normality test and equal variance test, all with 95% coefficient interval. One-way ANOVA was followed by Tukey (honest significant difference) post-hoc testing. A *p*-value ≤ 0.05 was accepted as statistically significant. 

Combination index (CI; value which determines multiple drug interaction) was calculated by the Chou–Thalaya method using CompuSyn software (ComboSyn, Inc.; Paramus, NJ, USA). Data for cell viability and cell inhibition measured by CyQUANT Direct was used to generate a dose-effect curve and a median-effect plot. Data were obtained for combination of non-constant ratio design. CI was calculated by the following formula (CI for mutually exclusive drugs):

(1) $$\mathrm{CI}=\frac{\left(D\right)1}{\left(\mathrm{Dx}\right)1}+\frac{\left(D\right)2}{\left(\mathrm{Dx}\right)2}$$

where D is the concentration of single drugs and Dx, a dose of D alone, which gives x inhibition. Drug synergism was accepted with CI < 1, additive effect with CI = 1, and antagonism with CI > 1.

Data acquired by flow cytometry (cell death Annexin V/PI) were further analysed by Flowing Software program (free-downloadable software developed by Perttu Terho, version 2.5.1; Turku, Finland). Distinctive gating was applied on cell population events to compensate for auto-fluorescence of Dox and back-gating was used to define cells that were double-stained with Annexin V and PI when analysing the cell population data.

## 3. Results

### 3.1. Combination of Betulinic Acid and Doxorubicin Synergistically Reduced Cell Viability in MOLM-13 AML Cell Line, but Did Not Significantly Affect the Viability of U-937 Cells

**Figure 1:**
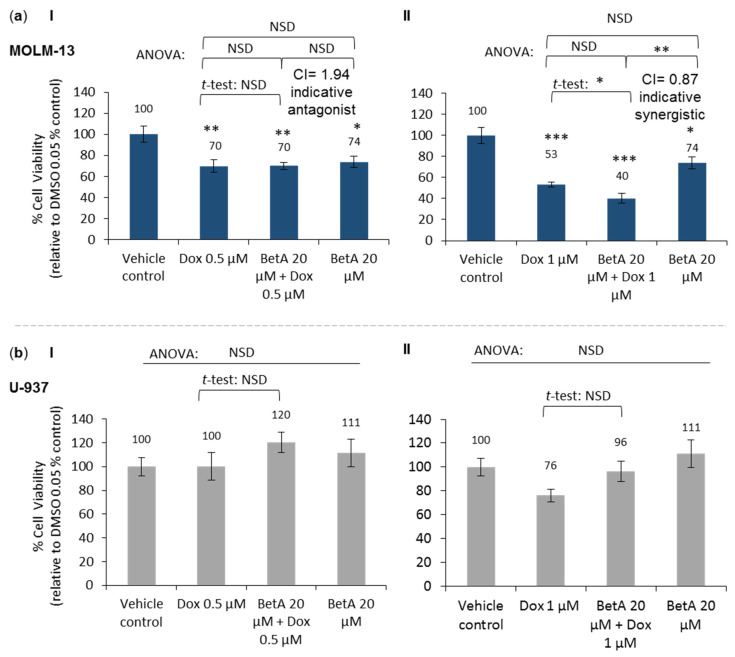
Effect on cell viability of betulinic acid and doxorubicin combination treatments on leukaemic MOLM-13 and U-937 cells MOLM-13 (**a**) and U-937 (**b**) cells were treated for 24 h by Dox 0.5 μM with BetA 20 μM (**I**) and Dox 1 μM with BetA 20 μM (**II**), and the respective individual drugs. The data were expressed as mean ± SE of percentage relative to values obtained for control cells (cells with DMOS 0.05% vehicle). Cell viability was determined by fluorescence using CyQuant Direct assay. The data set was analysed by one-way ANOVA with Tukey post-hoc test. Single Dox and combination drugs were compared by two samples *t*-test. *n* = 4. Statistical difference was accepted as following: *p* > 0.05 no significant difference (NSD), * *p* ≤ 0.05 significant, ** *p* ≤ 0.01 highly significant, *** *p* ≤ 0.001 very highly significant. Synergy quantification was based on Chou–Talaya method by combination index (CI) using CompuSyn (non-constant ratio design). Additive effect (CI = 1), synergism (CI < 1), antagonism (CI > 1).

Experiments were conducted to determine if BetA could enhance the cytotoxic effect of Dox death in a relapsed AML cell model (MOLM-13) and compare this effect to another AML cell (U-937). Sole treatments of Dox 0.5 μM and 1 μM significantly reduced the cell viability of MOLM-13 (*p* < 0.01 and *p* < 0.001, respectively (Figure 1a)), compared to vehicle control (DMSO 0.05%). A non-statistically significant effect was shown in U-937 monocytes treated by Dox (0.5 and 1 μM) at 24 h, despite the suppression of U-937 cell growth by 24% when treated with Dox (1 μM). BetA (20 μM) showed selective cytotoxicity by inhibiting MOLM-13 cell growth at 24 h by 26% (*p* < 0.05) without a growth inhibitory effect on U-937 (Figure 1a,b).

Co-treatment of BetA and Dox at 24 h showed selective cytotoxicity towards the MOLM-13 cell line (Figure 1a). BetA 20 μM combined with Dox 0.5 μM or Dox 1 μM showed highly significant cell growth inhibition in MOLM-13 by 30% (*p* < 0.01) and 60% (*p* < 0.001), respectively, at 24 h. No statistical effect on cell growth was observed on U-937 monocytes by the same combination treatments, despite an increase in cell numbers by 20% at BetA 20 μM and Dox 0.5 μM combination (Figure 1b). Therefore, combination treatments were cytotoxic to the leukaemic relapsed cell model but did not significantly affect the cell viability of monocytic U-937 cells.

Comparison of statistical mean difference between single drugs and combination treatments in MOLM-13 gave contradicting outcomes when using parametric statistical tests, which compares variances (ANOVA; post-hoc Tukey and two sample *t*-test). The effect of the combined drug was not statistically significant when compared to the sole treatment with Dox 1 μM based on ANOVA (*p* > 0.05), while significant (*p* < 0.05) with two sample *t*-test analysis (Figure 1(aII)). However, the Chou–Talaya method using combination index (CI) has been reported to be a more reliable way to interpret results for combinational studies over analysis of variances [41,42]. Based on the CI value, the inhibition effect of BetA combined with Dox 1 μM was indicative synergistic (CI < 1) in suppressing the viability of MOLM-13 at 24 h incubation. However, BetA combined with 0.5 μM Dox was indicative antagonistic (CI > 1) in their inhibitory effect. Although not showing the conventional antagonistic trend (increase in cell viability), the terminology ‘antagonist effect’ was used based on the combination index (CI) value generated by Chou–Thalaya method.

### 3.2. Combination Treatments Induced Apoptotic Death in MOLM-13 AML Cell Line, but Rescued U-937 Cells from Doxorubicin-Induced Cell Death

**Figure 2:**
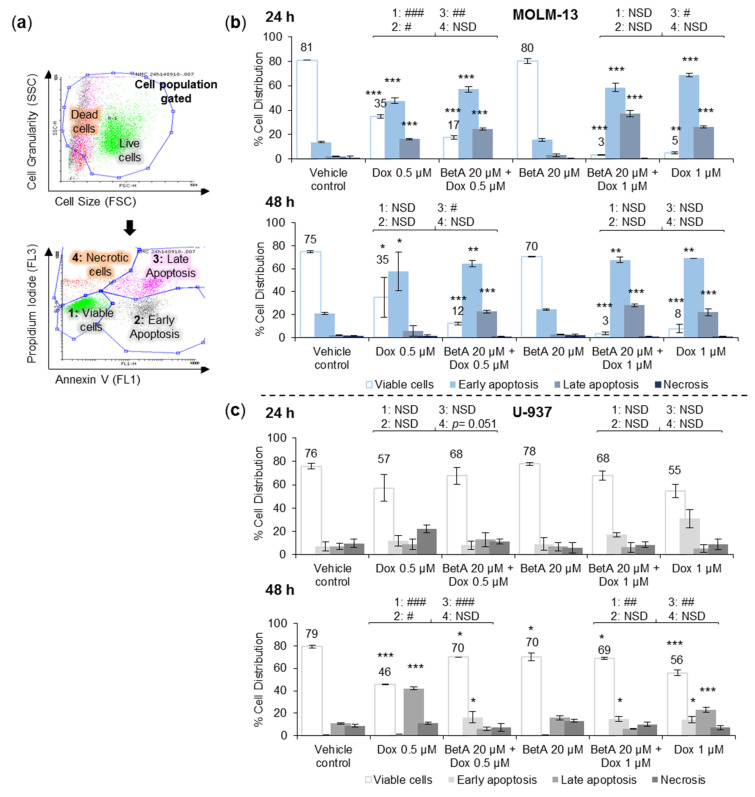
Cell death population of MOLM-13 and U-937 treated by individual drugs and combined therapy for 24 and 48 h. Samples were double stained by Annexin V (apoptotic cell dye) and Propidium Iodide (PI, dead cell dye). Gating of treated MOLM-13 and U-937 cells to determine cell death population (**a**). MOLM-13 (**b**) and U-937 (**c**) cells were treated with BetA (20 µM), Dox (0.5 and 1 µM), and BetA (20 µM) combination with Dox (0.5/1 µM) for 24 and 48 h incubation. DMSO 0.05% was used as vehicle cell control. One-way ANOVA; Tukey post-hoc test was used to compare the cell population between the vehicle control and the treatments. Shift in different cell death population between single Dox and combination was compared using two samples *t*-test analysis. *n* = 3. Statistical difference was accepted as following: *p* > 0.05 no significant difference (NSD), * *p* ≤ 0.05 significant, ** *p* ≤ 0.01 highly significant, *** *p* ≤ 0.001 very highly significant. Combination and single Dox cell population compared by two sample *t*-test: *p* > 0.05 (NSD), # *p* ≤ 0.05, ## *p* ≤ 0.01, ### *p* ≤ 0.001. 1: Live cells (−ve Annexin V and −ve PI), 2: Early apoptosis (+ve Annexin V and −ve PI), 3: Late apoptosis (+ve Annexin V and +ve PI), 4: Necrotic cells (−ve Annexin V and +ve PI).

Annexin V and Propidium Iodide (PI) double staining was used to examine the effect of drug-induced cell death and to determine the proportion of cells in different apoptotic stages (Figure 2a). Cell death was observed in single Dox treatments, as well as Dox and BetA co-treatments in MOLM-13 cell line at 24 and 48 h incubation with very high statistical differences (*p* < 0.001), compared to vehicle control. However, BetA (20 µM) alone induced similar apoptotic death profile compared to DMSO control with *p* > 0.05 at both time points (Figure 2b). Co-treatments of the combined drugs with MOLM-13 showed a slight shift in the ratio of distinct cell death population when compared to single Dox. At 24 h, about 10% (*p* < 0.05) more cells resided in reversible early apoptotic stage (+ve Annexin V, −ve PI) and 8% (*p* < 0.01) less cells in irreversible late apoptosis (+ve Annexin V, +ve PI) with Dox 0.5 µM, when compared to the equivalent Dox co-treatment with BetA. In addition, combination treatment of Dox 1 µM and BetA had significantly more (11%, *p* < 0.05) cells in late apoptosis compared to Dox 1 µM alone at 24 h. At 48 h, the lower combination (Dox 0.5 µM and BetA 20 µM) had significantly more cells (17%, *p* < 0.05) present in late apoptosis and less viable cells (23%) compared to 0.5 µM Dox (Figure 2b), however, it was not statistically significant. At 48 h, Dox 1 µM killed most of the MOLM-13 cells (92%), whereas Dox 1 µM and BetA combination induced 97% cell death. This difference was non-statistically significant. 

In U-937 cells at 24 h incubation, Dox treatments showed no statistical significance in cell population shift compared to the vehicle control cells, despite Dox 0.5 µM appearing to have more cells in necrosis (12%) and Dox 1 µM more cells in early apoptosis (24%). BetA (20 µM) alone showed no significant change in cell death induction or cell viability suppression when compared to the control, nor enhanced the cell killing ability of Dox treatments (Figure 2c). Combination treatments on average have more viable cells present compared to their single Dox counterparts (without significance). At 48 h incubation, cell death induced in U-937 cells by both concentrations of Dox (0.5 and 1 µM) was more pronounced compared to the control treatment (viable cell reduction; *p* < 0.001), with the majority of cells residing in late apoptosis (*p* < 0.001; 42% in Dox 0.5 µM and 23% in Dox 1 µM). Although, sole treatments of BetA in U-937 cells at 48 h showed statistically less viable cells (9%; *p* < 0.05) than the control, none of the cell death populations were significantly different. Moreover, BetA with Dox co-treatment attenuated Dox-induced cell death in U-937 cells by roughly 25% and 13% compared to equivalent single Dox 0.5 µM (*p* < 0.001) and 1 µM (*p* < 0.01), respectively (Figure 2c). 

Interestingly, the effect of Dox in terms of apoptotic population over 48 h did not show much variation. Although the incubation period did not have much impact on the concentrations of Dox tested, the overall ratio of cell population after Dox exposure remained steady, which was seen in both cell lines.

### 3.3. Betulinic Acid and Doxorubicin Combination Enhanced the Formation of Reactive Oxygen Species in MOLM-13 Cell Lines

**Figure 3:**
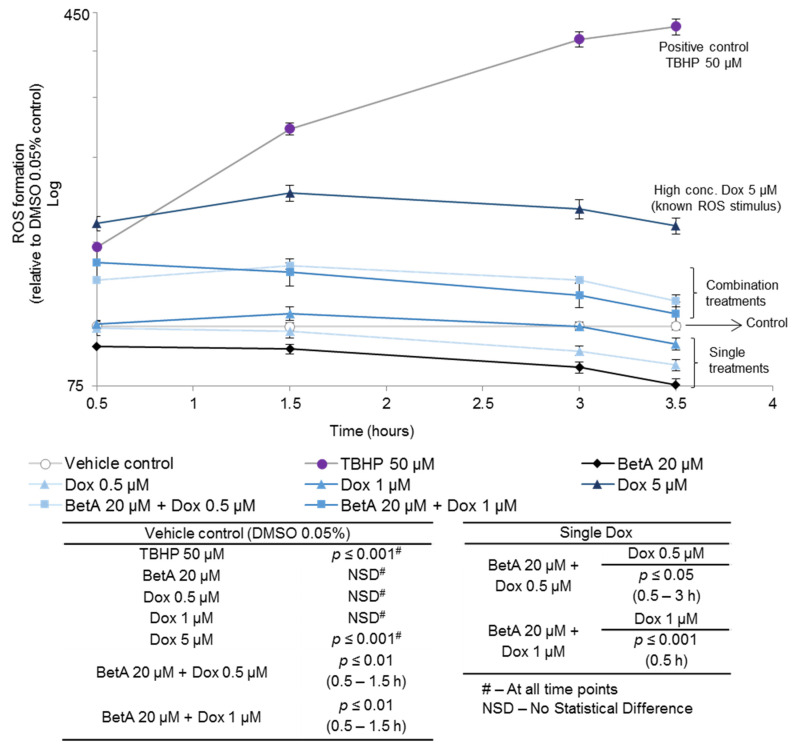
Reactive oxygen species stimulation in MOLM-13 after treatment with doxorubicin, betulinic acid and their combination. The cells were stained with 20 µM DCFDA following 0.5, 1.5, 3 or 3.5 h co-treatment with BetA (20 µM), Dox (0.5 or 1 µM) or a combination of the drugs. TBHP (50 μM) was used as a positive control. ROS stimulation was measured as the fluorescence of the treatments using FLUOstar Omega (BMG Labtech) at 485/520 nm, excitation and emission, respectively. Data are expressed as the mean ±SE of four replicate (*n* = 4) measurements in one representative experiment. The data were analysed by one-way ANOVA using Tukey post-hoc analysis. Dox and the combined treatments were compared by two sample *t*-test.

Reactive oxygen species (ROS) are associated with the intrinsic (mitochondrial) apoptotic cell death pathway. In this study, MOLM-13 cells individually treated with the positive control drugs, Dox (5 μM) or TBHP (50 μM), significantly (*p* < 0.001) stimulated ROS generation. Although single treatments of low dose Dox (0.5 or 1 μM) induced cell apoptosis in MOLM-13, they failed to exhibit an increase in ROS levels after treatment of MOLM-13 cells with the drugs for up to 3.5 h (Figure 3). Single BetA (20 µM) treatment did not significantly alter the levels of ROS when compared to the vehicle control. However, the combined treatments stimulated ROS production within 1.5 h after cell treatment (*p* < 0.001 when compared to vehicle control). In addition, the combination treatments significantly enhanced ROS generation when compared to single Dox treatments. BetA augmented Dox 0.5 µM and 1 µM ROS levels to 1.3–1.4-fold within 0.5–3 h (*p* < 0.05) and 1.4-fold at 0.5 h (*p* < 0.001), respectively.

### 3.4. Doxorubicin, Alone and in Combination with Betulinic Acid, Inhibits a Novel Isoform of Bcl-2 in AML MOLM-13 Cells without a Potent Effect on the Main Bcl-2 Isoform

**Figure 4:**
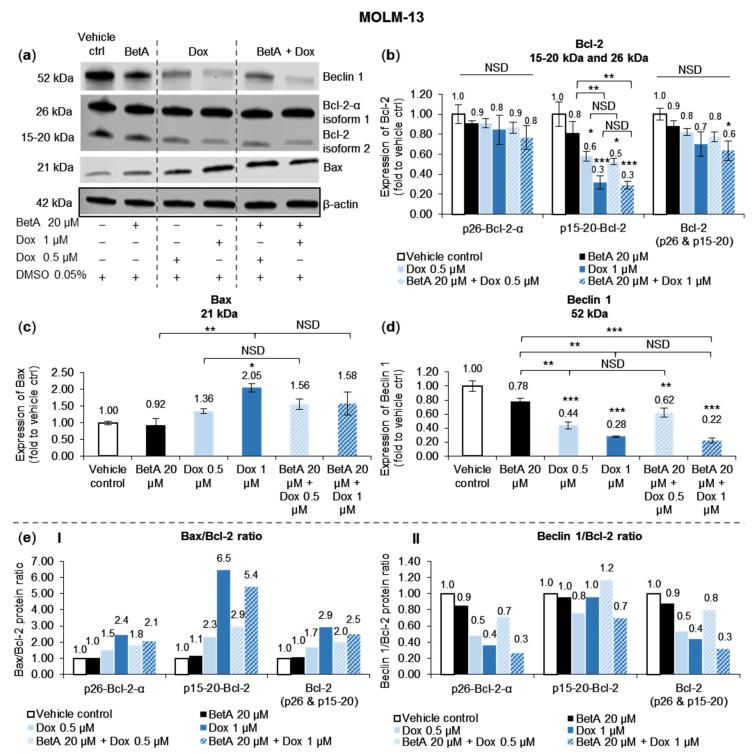
The effects of BetA, Dox and combination on apoptotic Bcl-2 family and autophagy Beclin 1 protein expressions on the MOLM-13 cell line. The protein expressions of BH3-containing proteins, autophagic Beclin 1 (52 kDa), anti-apoptotic Bcl-2 (26 and 15–20 kDa) and pro-apoptotic Bax (21 kDa) were estimated by Western blot (**a**). Experimental samples were run on the same gel and shown protein expressions are on the same blot for each protein antibody. Bcl-2 (**b**), Bax (**c**) and Beclin 1 (**d**), protein levels were determined by normalising quantified protein intensity relative to vehicle control (DMSO 0.05%, represented as 1.00-fold) and ratio between normalised data and β-actin (housekeeping protein). Data were expressed as mean + SE (*n* = 3) and analysed by one-way ANOVA; Tukey post-hoc test. Statistical difference was accepted as following: *p* > 0.05 no significant difference (NSD), * *p* ≤ 0.05 significant, ** *p* ≤ 0.01 highly significant, *** *p* ≤ 0.001 very highly significant. Relative change in the ratio (**e**) of Bax/Bcl-2 (**I**) and Beclin1/Bcl-2 (**II**) were expressed as the fraction of the mean protein levels.

Sole BetA 20 µM did not significantly alter the expression of Bcl-2 or Bax protein. Additionally, the effect of Dox on apoptosis-regulating proteins and the isoforms was not enhanced when combined with BetA. Therefore, BetA did not enhance but also did not interfere with the effect of Dox in regulating the pro- and anti-apoptotic proteins Bax and Bcl-2 in MOLM-13 (Figure 4).

The expression level of Bax pro-apoptotic protein was increased by single Dox and combination treatments, but this protein elevation was not statistically different between the treatments and vehicle control, except for 1 µM Dox treatment that increased Bax level by 2.1-fold (*p* < 0.05) (Figure 4c). The active Bax protein is antagonised when bound to Bcl-2 protein in a complex. Dox and combination treatments reduced the level of Bcl-2 anti-apoptotic protein in MOLM-13. However, the decrease was statistically significant only for the smaller Bcl-2 of 15–20 kDa (p15-20-Bcl-2) and not in the bigger 26 kDa Bcl-2 isoform (p26-Bcl-2-α), when compared to vehicle control. Therefore, the treatments potently affected the novel truncated p15-20-Bcl-2 reported by Vu et al. (2020) [22]. When the Bcl-2 isoforms were combined, statistical reduction (*p* < 0.05, compared to vehicle control) was shown only with the combination treatment of Dox 1 µM and BetA 20 µM (Figure 4b). 

To assess the susceptibility of the AML cells undergoing apoptosis, Bax/Bcl-2 ratio was evaluated (Figure 4(eI)). All treatments, except for sole BetA, increased the ratio in both Bcl-2 isoforms, with the Bax/Bcl-2 ratio of p15-20-Bcl-2 being much greater. Moreover, the ratio difference when comparing single Dox and the combination with BetA was similar regardless of the Bcl-2 isoform. Combination of Dox 0.5 µM and BetA 20 µM slightly enhanced the ratio more than single Dox 0.5 µM treatment: an increase by 1.2–1.3-fold. On the contrary, the ratio increase of Dox 1 µM and BetA 20 µM combination was marginally lower when compared to Dox 1 µM treatment, a reduction by 1.2-fold. 

### 3.5. Autophagy Marker Beclin 1 Was Reduced by Doxorubicin and Betulinic Acid Co-Treatment in AML Cell Line

Expression of Beclin 1 protein, an autophagy marker, was significantly decreased in MOLM-13 cells when treated with Dox alone (0.5 µM, *p* < 0.001 and 1 µM, *p* < 0.001) and Dox combined with BetA 20 µM (Dox 0.5 µM, *p* < 0.01 and 1 µM, *p* < 0.001) when compared to the expression of vehicle control (Figure 4d). The decrease of Beclin 1 protein was in a dose-dependent manner. Single BetA 20 µM slightly reduced the protein expression but with no statistical significance when compared to the control. In addition, there was no apparent effect of BetA on Dox activity in regulating the Beclin 1 expression. 

To evaluate autophagy–apoptosis regulatory pathway and explore its association with cell death, Beclin 1/Bcl-2 ratio was calculated. The ratio was dose-dependently reduced by Dox and combination treatments with the established p26-Bcl-2-α, but no marked change was shown with BetA treatment. Moreover, there was a pronounced reduction in Beclin 1/p26-Bcl-2-α by Dox and combination treatment, which was in contrast to the Beclin1/p15-20-Bcl-2 ratio where there was less reduction (Figure 4(eII)). The effect of the drugs (singly and combined) on the Beclin 1/Bcl-2 ratio across p26-Bcl-2-α, p15-20-Bcl-2, and combined isoforms (Bcl-2, p26 + p15-20) was similar. Dox 0.5 µM and BetA 20 µM combination marginally increased Beclin 1/Bcl-2 ratio by 1.5-fold compared to single Dox 0.5 µM. In contrast, combination of Dox 1 µM and BetA 20 µM slightly reduced the ratio by 1.4-fold compared to single Dox 1 µM treatment (Figure 4(eII)).

### 3.6. Apoptotic and Autophagy Signalling Protein Levels Were Not Altered by the Treatments in U-937 Cells

**Figure 5:**
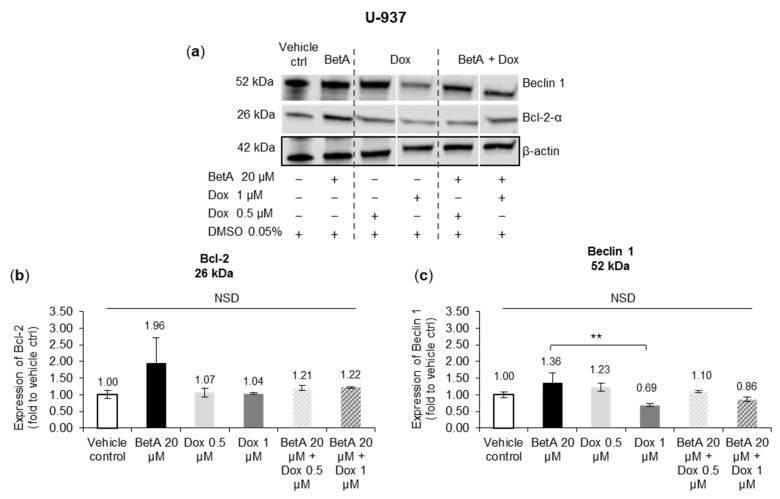
Apoptosis and autophagy regulating proteins expression in U-937 monocytes treated by single and combination drug. Leukaemic cell line U-937 cells were treated by single Dox or combination of BetA and Dox for 48 h. The protein expressions of autophagy marker Beclin 1 (52 kDa) and anti-apoptotic Bcl-2 (26 kDa) were estimated by Western blot (**a**). Experimental samples were run on the same gel and shown protein expressions are on the same blot for each protein antibody. Bcl-2 (**b**) and Beclin 1 (**c**) protein levels were determined by normalising quantified protein intensity relative to vehicle control (DMSO 0.05%, represented as 1.00-fold) and ratio between normalised data and β-actin (housekeeping protein). Data were expressed as mean + SE (*n* = 3) and analysed by one-way ANOVA; Tukey post-hoc test. Statistical difference was accepted as following: *p* > 0.05 no significant difference (NSD), ** *p* ≤ 0.01 highly significant.

In U-937 monocyte cells, none of the treatments altered significantly the expression levels of anti-apoptotic Bcl-2 after 48 h treatment when compared to the vehicle control. Furthermore, only one isoform of Bcl-2 (p26-Bcl-2-α) was expressed in U-937 cells and there was no detection of any other isoforms such as p15-20-Bcl-2 that was reported in MOM-13 cells (Figure 5a). BetA-treated U-937 cells showed on average 1.96-fold increase in Bcl-2 (26 kDa) expression, but without significance (Figure 5b). 

Beclin 1 expression level was not significantly changed by any tested treatments in U-937 cells when compared to vehicle-treated cells. Although, U-937 cells treated by single Dox 1 µM marginally declined Beclin 1 protein level (0.7-fold; *p* > 0.05 compared to vehicle control), which was significantly (*p* < 0.01) lower compared to BetA 20 µM treatment that slightly increased Beclin 1 expression (1.4-fold; *p* > 0.05 compared to vehicle control) (Figure 5c).

### 3.7. Combination Treatment Altered mRNA Expression of Bcl-2 Family Members and Autophagy towards Cell Death in MOLM-13 Cells, but Survival in U-937 Cells

Alteration in gene level could potentially be associated with the protein expression change as transcription and translation is tightly linked. Therefore, the gene transcript (mRNA) was quantified to further examine the mechanism of action and the pathways involved in cell death induction by the drugs.

**Figure 6:**
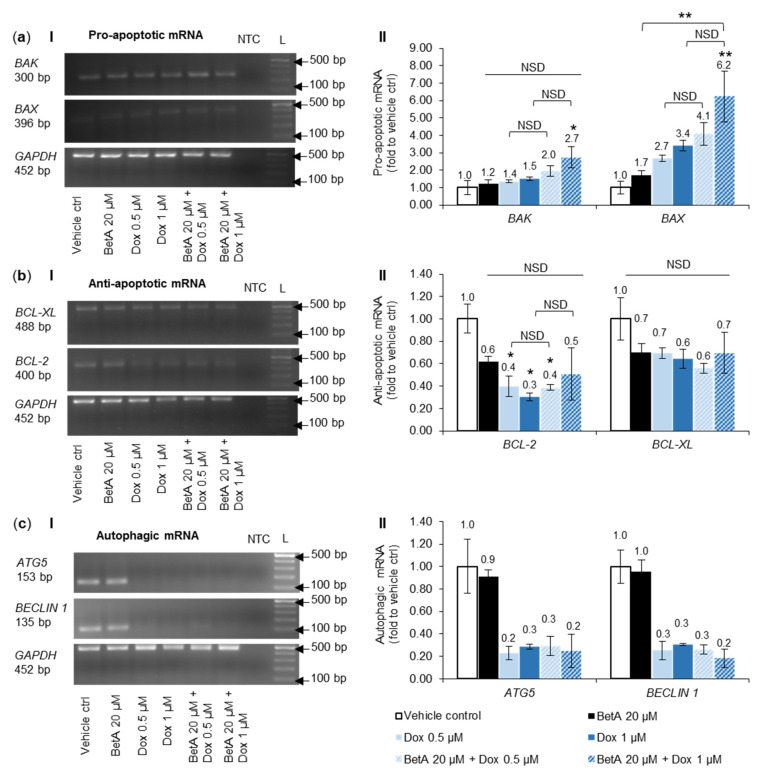
Expression of apoptotic Bcl-2 family members and autophagy genes in MOLM-13 cell lines co-treatment with single and combination drugs after 48 h. RT-PCR gene expression detection by gel electrophoresis (**I**) of MOLM-13 treated cells were quantified (**II**) using ImageJ software. The mRNA expression of pro-apoptotic (**a**) BAK and BAX (*n* = 3), anti-apoptotic (**b**) BCL-2 and BCL-XL (*n* = 3), and autophagy (**c**) ATG5 and BECLIN 1 (*n* = 2) is relative to vehicle control (DMSO 0.05%, represented as 1.00-fold) and ratio between normalised data and GAPDH (housekeeping gene). NTC—non-template control. L—molecular ladder PCRBIO Ladder IV (100–1500 bp). Results were expressed as mean + SE. The data set was analysed by one-way ANOVA; Tukey post-hoc test. Statistical difference was accepted as following: *p* > 0.05 no significant difference (NSD), * *p* ≤ 0.05 significant, ** *p* ≤ 0.01 highly significant.

Dox and BetA alone marginally, but not significantly, increased the mRNA expression of pro-apoptotic BAK and BAX genes in MOLM-13 cells compared to vehicle control. Combination treatments further increased the mRNA of pro-apoptotic proteins when compared to the control, but only the combination with higher Dox concentration (BetA 20 µM + Dox 1 µM) significantly upregulated both BAK (2.7-fold increase, *p* < 0.05) and BAX (6.2-fold increase, *p* < 0.01). However, the effect was not significantly different when compared to the equivalent single Dox treatment (Figure 6a). 

Anti-apoptotic BCL-XL mRNA expression was not statistically changed by any of the tested drugs in MOLM-13 cells, there was no significant difference between treatments and the vehicle control. Conversely, the anti-apoptotic mRNA BCL-2 expression was downregulated by the drug treatments. Single Dox treatments dose-dependently reduced (*p* < 0.05) BCL-2 expression by more than half in MOLM-13 cells, while BetA alone also showed a decline (on average by 0.62-fold relative to vehicle control) but with no statistical significance. Although both combination drug treatments with MOLM-13 cells reduced (on average) the BCL-2 level, it was significant (*p* < 0.05) only with BetA 20 µM and Dox 0.5 µM combination. In addition, there was no difference in the mRNA expression between single drugs and combination treatments (Figure 6b).

The band expression of autophagy genes ATG5 and BECLIN 1 (ATG6) was clearly detectable in MOLM-13 cells treated by the vehicle control and single BetA treatment. BetA 20 µM alone did not change the regulation of ATG5 and BECLIN 1 genes in MOLM-13 cells, from vehicle control expression. Conversely, ATG5 and BECLIN 1 mRNA expression in MOLM-13 cells were markedly downregulated to almost undetectable levels by single Dox treatments and combination treatments compared to vehicle control levels (Figure 6c). 

**Figure 7:**
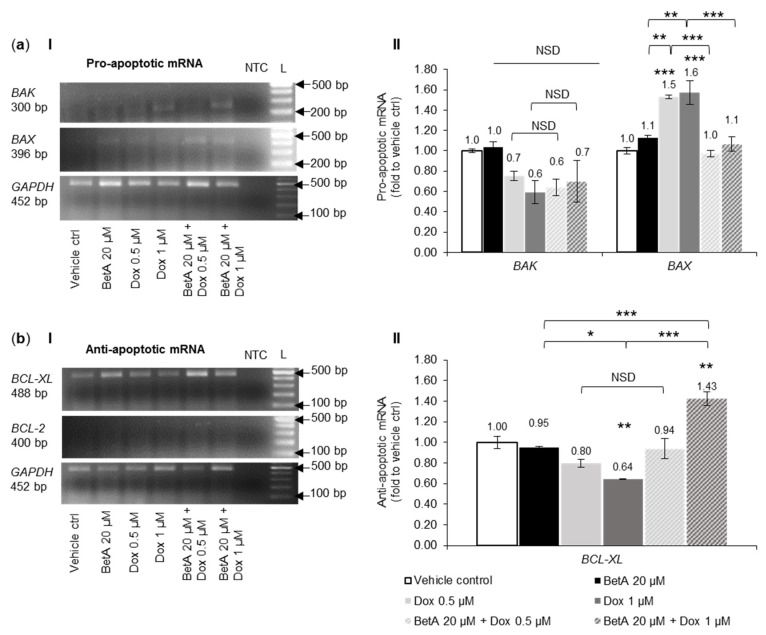
Expression of pro- and anti-apoptotic Bcl-2 family member genes in U-937 cells after co-treatment with single and combination drug for 48 h. Gene expression by gel electrophoresis (**I**) of U-937 treated cells quantified (**II**) using ImageJ software. The mRNA expression of pro-apoptotic (**a**) BAK and BAX (*n* = 3) and anti-apoptotic (**b**) BCL-2 and BCL-XL (*n* = 3) is relative to vehicle control (DMSO 0.05%, represented as 1.00-fold) and ratio between normalised data and GAPDH (housekeeping gene). NTC—non-template control. L—molecular ladder PCRBIO Ladder IV (100–1500 bp). Result were expressed as mean + SE. The data set was analysed by one-way ANOVA; Tukey post-hoc test. Statistical difference was accepted as following: *p* > 0.05 no significant difference (NSD), * *p* ≤ 0.05 significant, ** *p* ≤ 0.01 highly significant, *** *p* ≤ 0.001 very highly significant.

In U-937 monocytic cells, no significant change in the expression of pro-apoptotic BAK mRNA was observed between the treatments and the vehicle control. The pro-apoptotic BAX was significantly (*p* < 0.001) upregulated in U-937 by single Dox treatments, but single BetA 20 µM did not change the expression when compared to vehicle control (Figure 7a).

Interestingly, the effect of Dox treatments (0.5 and 1 µM) on BAX upregulation was reduced to the expression level of vehicle control when combined with BetA 20 µM in U-937 cell co-treatments. The difference in BAX mRNA between single Dox and their relevant BetA combination was very highly significant (*p* < 0.001) (Figure 7(aII)). 

Anti-apoptotic BCL-2 mRNA expression was not detected in U-937-treated cells when the same experimental conditions that were used for other genes and different cells were applied (Figure 7(bI)). Anti-apoptotic BCL-XL was dose-dependently downregulated in U-937 monocytic cells by single Dox treatments with statistical significance (*p* < 0.01) at 1 µM. The mRNA levels of BCL-XL were not changed in U-937 cells treated by BetA 20 µM alone when compared to vehicle-treated cells. However, BetA 20 µM upregulated BCL-XL expression of U-937 cells when co-treated with Dox 1 µM. This combination treatment hindered the significant downregulating effect of Dox 1 µM and significantly increased (*p* > 0.001) regulation of BCL-XL mRNA level (Figure 7(bII)).

## 4. Discussion

Standard chemotherapy drugs such as Dox can potently eliminate AML cells, however, they also exert toxicity on non-transformed/non-malignant cell lines [5]. In addition, AML is prone to cell relapse leading to production of more refractory cells that may develop resistance to chemotherapy and avoid apoptosis [43]. In our recent publication we reported that Dox exhibited selectivity toward relapsed AML cells, associated with reduction of p15-20-Bcl-2 and Beclin 1 protein expression in MOLM-13 cells [22]. 

Based on our preliminary studies, and reports in the literature, a concentration of 20 µM BetA was used in combination studies with clinically relevant concentrations of Dox (0.5 and 1 µM) in the currently reported study. Reactive oxygen species generation, pro- and anti-apoptotic Bcl-2 family and autophagy proteins, as well as gene involvement in apoptotic death and autophagy modulation by BetA, Dox, and their combination were examined. 

### 4.1. Cytotoxic Effect of Betulinic Acid on Leukaemia Cell Lines

Anticancer effects of BetA have been studied mostly in cancers of epithelial origin [5,7,10,44,45,46,47] with only limited studies on blood cancers. BetA has been reported to cause a reduction of leukaemic cell viability in chronic myelogenous leukaemia (CML) cell line, K562 [9,48] and in HL-60 (human promyelocytic leukaemia cells) [49]. In our study, sole treatment of BetA suppressed cell viability of MOLM-13 cells (Figure 1a). However, this effect was not seen in another AML cell line, U-937, with the same BetA concentration (Figure 1b). Thus, BetA displayed some selectivity in MOLM-13 cells. However, it is not clear why BetA exhibited selectivity in MOLM-13 cells—these cells are derived from a relapsed patient.

Studies comparing human primary melanocytic (non-cancerous) and melanoma cells showed that the normal (non-cancerous) cells were less susceptible to BetA [2]. In another study, BetA demonstrated minimal cytotoxic effect in normal colon cells while potently inhibiting the growth of colorectal carcinoma (HCT 116) cells in a dose-dependent manner [3]. In normal blood cells, BetA tested up to a high dose of up to 110 μM on peripheral blood lymphoblast [5] and 66 μM on peripheral blood mononuclear cells [4] did not affect or suppress cell growth. Thus, evidence from the literature indicates that BetA potentially targets cancerous cell lines, which is a desirable compound characteristic when treating malignant cells. 

### 4.2. The Effect of BetA-Dox Drug Combination on Cancer Cell Viability

BetA combinations with other forms of therapy have shown some anti-cancer potential. For example, BetA showed an additive effect in combination with irradiation therapy in melanoma cells [2]. Studies co-treating BetA with other phytochemicals such as α-Mangostin [3] or ginsenoside Rh2 [50] showed enhanced cytotoxicity and apoptotic death in cancer cell lines of epithelial origin. Another study combined BetA with TRAIL (tumour necrosis factor-related apoptosis-inducing ligand) and demonstrated enhanced apoptotic activity in various cell lines (neuroblastoma, medulloblastoma, glioblastoma and melanoma) [51]. Therefore, in this study, BetA was selected to be tested with established AML chemotherapy drug Dox.

Combination of BetA (20 µM) and anthracycline chemotherapy drug Dox (1 µM) significantly reduced cell viability of MOLM-13 compared to single Dox 1 µM treatment, the interaction between the compounds was indicative synergistic (CI < 1) in this study (Figure 1(aII)). Fulda and Debatin (2005) [6] have hypothesised that BetA may sensitise tumour cells to chemotherapy drugs and the authors postulated that this could be due to combined drugs amplifying weaker death signals in apoptosis, leading to enhanced induction of cell death. However, it must be taken into consideration that the anthracycline class of drugs can block transcriptional induction and thus may potentially block members of the pro-apoptotic Bcl-2 proteins stimulated by other therapies. For instance, Dox was shown to be able to rescue cancer cell lines from cell-induced death by bortezomib and vorinostat [52]. Therefore, it is essential to assess multiple drug effect interactions (by combination index (CI)) of the combination therapy that utilises the anthracycline class of drugs. 

### 4.3. Betulinic Acid Enhanced Anticancer Drug Activity of Doxorubicin by Sensitising the Cancer Cell Lines to Apoptosis and ROS Formation

In this study, combination treatment of BetA and Dox hampered Dox-induced apoptosis in U-937 cells (Figure 2c). The selective effect of the combination treatment between the leukaemic monocytic cells may provide some insight on targeting cells. However, more work is required to determine how BetA differentiates and targets specific cell types. Thus, studies on cell death mechanism of action should further proceed to determine the difference between the molecular pathway inductions in a wider panel of cell lines.

BetA and Dox combination induced apoptotic death in the combination treatments through an increase of late apoptotic population in MOLM-13 cells (Figure 2b), which is more irreversible in relation to cell death [53]. A similar study by Fulda and Debatin (2005), experimented on neuroblastoma cells and tested co-treatments of BetA (6.6, 8.8 or 10.9 μM) and Dox (0.05, 0.2 or 0.4 μM) which triggered cell death in a dose- and time-dependent manner. The authors determined the induced cell death to be apoptotic based on the presence of DNA fragmentation, elevation of Smac (mitochondria-derived caspase activator), Cytochrome c release from mitochondria and activation of caspase-8, -3 and poly-ADP-ribose polymerase (PARP) [6].

The PARP family of proteins respond to DNA damage and are involved in maintaining genomic stability [54]. Cellular insults such as an increase in ROS formation have been reported to activate PARP, as ROS is known to cause oxidative DNA lesions [55]. ROS are by-products of normal cellular metabolism and in small amounts are necessary for maintaining cellular homeostasis [56]. However, cancer cells are known to have elevated ROS levels, which contribute to its tumorigenesis [57]. Therefore, an altered redox environment in cancer cells makes them more sensitive to ROS/redox manipulation [56,57]. Disproportional formation of ROS in cancer cells is linked to mitochondrial membrane deregulation; this mitochondrial damage causes several events leading to apoptosis [55]. An increase in ROS is also associated with other modes of cell death pathways [58]. We have also previously reported that sole treatment of chronic myeloid leukaemic cell line, K562, by the antioxidant phytochemical known as baicalein induced cell death via induction of ROS [59]. The duality of antioxidants possessing pro-oxidant properties in cancer cells requires further studies. In the current study, single BetA had no significant effect on ROS production in AML MOLM-13 cells (Figure 3). This is in contrast to other reports where BetA was capable of ROS stimulation in other cancer cell lines, which the authors linked to the compound’s ability to induce cellular death associated with mitochondrial membrane permeabilisation [1,7,8,9,12,46,60,61,62]

Dox is a known ROS inducer. However, only supraclinical concentrations are capable of showing this effect [63,64] which is in line with this study; only Dox 5 µM significantly increased ROS (Figure 3). Although, BetA and low Dox concentrations alone did not alter ROS levels in MOLM-13, the compounds together significantly elevated ROS stimulation in MOLM-13 cell lines, showing a potential for mutual collaboration in the mechanism of action (Figure 3). Single BetA may have not shown a direct effect on ROS generation, but it could have sensitised the cells to enhance Dox-induced cell death through changing ROS susceptibility in the cells. According to a study by Acésio et al. (2016) BetA and Dox co-treatments have been shown to potentiate DNA damage, leading to an increase in programmed cell death activation [65]. This similar effect possibly led to the rise in apoptotic death of MOLM-13 cells, where combined drugs increased the late apoptotic cell population, as shown in this study. 

### 4.4. Bcl-2 Protein Family Regulation by the Combination Treatment in Apoptotic Cell Death

Overall, the differential expressions of anti-apoptotic and pro-apoptotic Bcl-2 family of proteins in cells dictates the induction of intrinsic apoptotic pathways by regulating permeability of the mitochondria outer membrane. A shift in their balance within the cells can activate downstream caspases, the key effectors of apoptosis [34,66].

From the literature, the involvement of Bcl-2 family in BetA-induced cell death is not conclusive [7,10,11,12,67] and in this study, no statistical effect was noted. The regulation of Bax protein expression was only upregulated significantly (*p* < 0.05) by sole treatment of Dox and this effect was not affected by the addition of BetA to Dox (Figure 4c). Other studies have also reported that pro-apoptotic proteins such as Bax, Bak [19,68] and Bid [69] are upregulated and activated upon Dox treatments in certain cancer cell lines. Expressions of mRNA, quantified as mRNA transcription, are closely associated with the protein translation [70]. Altered expression of genes encoding for various Bcl-2 family proteins have been documented in many human cancers including leukaemia [71]. Consistent with the results of the protein expression in this study, when Dox was combined with BetA, there was a significant (*p* < 0.05) upregulation of mRNA expression of pro-apoptotic BAX, as well as BAK in MOLM-13 cells (Figure 6a). However, the drug combination appeared to defend U-937 cells from Dox-induced pro-apoptotic BAX gene expression (*p* < 0.001) (Figure 7a) and increased (*p* < 0.01) anti-apoptotic BCL-XL at the mRNA level. The difference in the regulation between MOLM-13 and U-937 monocytic cells by the drug combination indicates some targeting potential. This corroborates the results from the live–dead experiments, where Dox-induced apoptotic death in U-937 cells was alleviated with BetA combinations (Figure 2c).

Caution must be exercised to infer that ROS is involved in the modulation of apoptotic Bcl-2 family (Bax and Bcl-2) and autophagic Beclin 1 (BH3-containing) protein expressions. The possible synergism and enhanced cell death reported are based on cell viability and cell death assessments, respectively. ROS activity can affect the fragile redox environment in cancer cells and thus can distress mitochondria directly [57]. Therefore, ROS could be a mechanism that was independent from the Bcl-2 family of protein regulation in affecting the mitochondria. Although BetA alone did not directly affect ROS regulation, the compound could have primed the cells to make them susceptible to ROS, thus sensitising cells to Dox activity.

Since activated Bax migrates from the cytosolic space to mitochondrial membrane [72], other investigative work on Bax localisation could provide a better understanding of its activation and involvement in cell death induced by the compounds. The ratio of Bax/Bcl-2 was only weakly different between the single Dox and combination treatments. Therefore, there may be other underlying mechanisms, not associated with Bcl-2 family regulation that contribute to the enhanced cell death induction by the combination treatment. In addition, understanding the role and function of the Bcl-2 isoforms specifically present in some cancer cells may be useful for selective cell targeting leading to cell eradication as well as modulation of several cell pathways.

### 4.5. Modulation of Autophagy upon Exposure by Betulinic Acid, Doxorubicin and Drug Combination

Increases in autophagy protein markers and subsequent autophagy activation is sometimes associated with chemotherapy treatment. The reason is not clearly defined but it could be a cell response to drug-induced stress. Therefore, if autophagy acts as a survival response against chemotherapy drugs, cancer cells can potentially utilise the process for pro-survival and tumorigenic function [73]. Studies have shown that Beclin 1, an initiator of autophagy, can be upregulated [74] or downregulated [75,76] by Dox. We report that Dox induced cell death in MOLM-13 cells, with a decrease in the expression of Beclin 1 protein (Figure 4d). This is in agreement with gene regulation where autophagy markers (ATG5 and BECLIN1) were not amplified in Dox- and combination-treated MOLM-13 cells, indicating downregulation (Figure 6c). When tested in U-937 cells, none of the drugs tested in this study had a significant effect on Beclin 1 expression (Figure 5c). Therefore, modulatory effects of Dox on Beclin 1 protein expression appear to be context and cell-dependent. 

BetA 20 µM concentration tested in this study did not show any considerable effect on Beclin 1 regulation in leukaemic MOLM-13 (Figure 4), nor was the protein affected by Dox. However, a study by Yang et al. (2012) demonstrated a decrease of Beclin 1 protein using higher concentrations of BetA (33 µM and above), which inhibited autophagic flux and induced apoptosis in the myeloma cell line, KM3 [77]. 

Beclin 1 is a BH3-containing protein that can be negatively regulated by anti-apoptotic Bcl-2 protein and is activated by dissociating from the Beclin 1:Bcl-2 complex [33,34]. The ratio between autophagic Beclin 1 and anti-apoptotic Bcl-2 determines sensitivity to autophagy stimuli [78,79]. Thus, an increase in the ratio denotes autophagy activation [78]. In this study, Beclin 1/Bcl-2 ratio was decreased in MOLM-13 cells by the treatments (single Dox and combination drugs) (Figure 4(eII)), which may suggest inhibition of the autophagy process but the drop in the ratio was more prominent with the usual p26-Bcl-2-α and negligibly with p15-20-Bcl-2. This interplay between Bcl-2 protein family and the autophagy effectors at mitochondrial site can regulate the machinery of both apoptosis and autophagy, interfering with cellular metabolism and cell responses [32]. 

Detection of autophagic flux would give a better indication of autophagy completion, which measures the degradation rate of the autophagy process [80]. The upregulation of autophagic flux is represented by the enhancement of LC3 conversion (from I to II) and decrease of p62 (reporter of ubiquitinated protein degradation) protein levels, based on the guidelines to interpret autophagy [77,81]. Moreover, further investigations beyond marker expressions presented in this study, and including specific markers are required to assess the nature of this potential autophagy modulation.

## 5. Conclusions

The combination treatment of BetA and Dox showed indicative synergistic cytotoxic effect on AML MOLM-13 cell lines. BetA in the drug combination did not affect Dox-induced death regulation by Bcl-2 family and Beclin 1. However, increases in ROS generation by the combination treatment could be a mechanical factor that enhanced cell death, resulting in drug synergism in MOLM-13 cells, which led to an increase in more cells undergoing late apoptosis. On the contrary, this combination treatment hindered the Dox apoptotic effect in AML cell line U-937 by salvaging cell viability, which was associated with the mRNA regulation of Bcl-2 family. Thus, the effect of the compounds together induced cell death as well as cell-dependent protective effects.

Studies are currently underway, and they include work with specific inhibitors targeting apoptosis and autophagy to determine the interplay between the two processes in cell death induction. Future work will also include knock-in and knock-out experiments on MOLM-13, U-937, and other cells to verify the role of the p15-20 protein in drug-treated cells. The localisation of marker proteins within cells will also be studied. Additionally, work is required to verify if the enhanced ROS generation, observed by the drug combination, could have contributed to the synergistic effect that augmented apoptotic activity through direct mitochondrial membrane disruption. 

## Figures and Tables

**Table 1:** Primer design for apoptotic Bcl-2 family and autophagy genes.

Gene	Accession Number	Primer Sequence (5′–3′)	Product Size (bp)
Pro-apoptotic Bcl-2 family			
BAK	NM_001188.4	Fwd: CTGTTTTTACCGCCATCAGCAGG	249
		Rev: CTCTCAAACAGGCTGGTGGCAATC	
BAX	NM_001291428.2	Fwd: CCGTTCATCTCAGTCCCCTG	396
		Rev: GAAGTGTGTCCCGAAGGAGG	
Anti-apoptotic Bcl-2 family			
BCL-2	NM_000633.3	Fwd: GACTTCTTCCGCCGCTACCG	341
		Rev: GACAGCCAGGAGAAATGAAAC	
BCL-XL	NM_138578.3	Fwd: CCCAGAAAGGATACAGCTGG	488
		Rev: GCGATCCGACTCACCAATAC	
Autophagy marker			
ATG5	NM_004849.4	Fwd: TCTAAGGATGCAATTGAAGCTCA	153
		Rev: GGCCCAAAACTGGTCAAATCT	
BECLIN 1	NM_003766.5	Fwd: GCTGGAAGACGTGGAAAAGA	135
		Rev: TCCAGCTGCTGTCGTTTAAATT	

Fwd—forward primer, Rev—reverse primer.

## Data Availability

The data presented in this study are available in article.
